# Assimilation and ethnic boundaries in Southern Thailand: exploring the Malay Muslim cultural resistance

**DOI:** 10.3389/fsoc.2026.1775605

**Published:** 2026-05-01

**Authors:** Muhammad Khalid Wardana, Bintar Mupiza, Astri Wulandari, Muhammad Rio Fariza

**Affiliations:** 1 Department of Social Science and Development, Faculty of Social Sciences, Chiang Mai University, Chiang Mai, Thailand; 2 School of Communication and Social Sciences, Telkom University, Bandung, Indonesia

**Keywords:** cultural resistance, deep south, ethnic boundaries, insurgency, Malay Muslim, Thailand peace process

## Abstract

In Thailand’s Deep South, insurgency remains an urgent issue which has not been resolved since 2004. Muslim cultural resistance in Thailand’s Deep South is rooted in resentment from state-driven assimilation, which has contributed partially to the insurgency. The method is drawing on qualitative data collected through short-term fieldwork observations conducted between April and May 2022 and in-depth interviews with 14 Malay Muslim key informants, primarily in Pattani Province. This study employs the concept of ‘ethnic boundaries’ to analyse how cultural practices function as a form of resistance. By foregrounding cultural resistance and ethnic boundary-making, this article contributes to the literature on the Deep South peace process, which has thus far insufficiently addressed the ethnic and cultural dimensions of Malay Muslim participation and influences. The findings show that Malay Muslim cultural resistance plays a crucial role in sustaining ethnic identity by maintaining clear boundaries that distinguish Malay Muslims from the Thai national identity, including the category of ‘Thai Muslim’. However, such cultural expressions are often viewed with suspicion by the Thai state, partly due to their perceived association with the ongoing insurgency in the Deep South.

## 1 Introduction

The Deep South is a designation used to collectively refer to Thailand’s three southernmost provinces—Pattani, Yala, and Narathiwat—that border Malaysia. These provinces are well-known due to longstanding insurgency in the region and have been continually governed under martial law of the Thai state since July of 2005 (Chalermsripinyorat, 2020; Keyes, 2010; Korinek et al., 2025; Nonnarong and Chokprajakchat, 2018; Panjor, 2024; Sateemae et al., 2015; Suwardi and Chambers, 2023; Feigenblatt, 2010). Cultural and historical factors explain why the insurgency continues in this area. A prominent cultural factor is that the Malay Muslims who form the largest proportion of the population in Thailand’s Deep South have a distinctive ethnic identity that contrasts with that of other citizens of the Thai state, including other Muslims. A historical factor is ancestors of today’s Malay Muslims living in the Deep South had their own sovereign, independent nation-state known as the Patani Kingdom, which was established in the 15th century. The annexation of Patani by Thailand in 1786 removed local sovereignty, while the region’s integration into the Thai state in 1909 put an end to its independence (Amir, 2013; Arphattananon, 2018; Askew, 2015; Boon, 1996; Chinda et al., 2024; Efferi et al., 2025; Gumilar et al., 2025; Hara, 2019; Pitsuwan, 1987; Rahman et al., 2025; Sateemae et al., 2015; Yong, 2023a, 2023b; Yusuf and Thongin, 2025).

Three main ethnics live in Deep South: Thai, Malay, and Chinese. In the beginning, the relationship between Patani and Thai kingdom were more like a power and territory bargain in process, as in the Mandala system (Puaksom, 2008). Historically, the Deep South is some of the oldest trading settlement place, where the intermarriage between Chinese and Thai creates a new Peranakan Chinese of Patani (Teo, 2003). After the fall of Patani kingdom in 1785, the Thai government dispersed its remaining powers by creating 7 districts (monthon in Thai language) of Patani territory (Puaksom, 2008). When Patani fall, the Peranakan Chinese fled from Patani, some reached Kelantan in Northern Malaysia. Since then, the relationship between Thai Buddhist and Malay Muslims worsen. The assimilation policies introduced in the 1930 further create a gap between both ethnics. An approach by Prime Minister Prem Tinsulanond in 1980’s reduced the tension between Thai Buddhist and Malay Muslim, yet the resentment felt by the Malay Muslim still exist. In the present days, the relationship between the three ethnics are relatively safe in terms of civilian relationship, yet the insurgency creates a sense of distrust between the Malay Muslim and the Thai Buddhist.

Central to the insurgency in the Deep South is the Malay Muslim struggle to have their identity acknowledged by the Thai government. Although Malay Muslims constitute a majority in the Deep South, they face culturally-suppressive policies from the central Thai government, which threaten the existence of their Malay heritage identity. The first struggle regarding their Malay identity when the Thai government began introducing assimilation policies that are incompatible with the traditional Malay Muslim way of life, such as the Buddhism Education Act, in the 1920s. Furthermore, during the second World War, Prime Minister Plaek Phibunsongkram introduced a Thai nationalist policy known as Ratthaniyom which restricted Malay identity and cultural expression (Albritton, 2010; Arphattananon, 2018; Charoenvattananukul, 2022; Efferi et al., 2025; Forbes, 1982; Haque, 2018; Jitpiromsri et al., 2020; Jory, 2007; Larsson, 2023; Pitsuwan, 1987; Feigenblatt, 2010; Yong, 2014, 2023a).

In response to the imposition of Thai assimilation policies, Malay Muslim formed a critical resistance movement. The first resistance to Thai authority such as the Patani People’s Movement (PPM, established in 1947) (Melvin, 2007). One of the early significant figures of the Malay Muslim resistance movement was Haji Sulong bin Abdul Kadir. Haji Sulong was well-known for his seven-point demands made to the Thai government in 1948 calling for “Malay privilege,” such as the right to use Malay language and practice traditional Islamic law in the Deep South (Ockey, 2011; Yong, 2023a, 2023b). However, the demands were not fully accepted, and his disappearance in August 1954 led to more unrest, worsening socio-political tension between the central Thai government and the local Malay Muslim population (Liow, 2006; Liow, 2006). Since then, numerous armed insurgent groups have risen which exacerbate the situation in Deep South (Albritton, 2010; Buranajaroenkij and Hayes, 2025; Chinda et al., 2024; Connors, 2006; Korinek et al., 2025; Rahman et al., 2025; Roul and Romaniuk, 2024; Sahapattana and Cheurprakobkit, 2019; Sateemae et al., 2015; Feigenblatt, 2010).

The Malay Muslim population today still faces challenging conditions regarding expression of their culture and identity. One of the challenges is the Thai government still views the cultural demands of the Malay Muslims as equivalent to their political aspirations for separatism from the Thai state (Melvin, 2007). Moreover, due to the difference between the Thai national identity and the Malay Muslim identity, Duncan McCargo (2011), explains that Malay Muslims are compelled to undergo a “*dual-track process*” to gaining full citizenship. The first is formal citizenship recognition using Thai identification cards as proof of citizenship. The second is the process for informal citizenship, which refers to recognizing an individual or group based on “attitude, self-presentation, and behavior”. However, as the Malay Muslim aspire to express their culture and identity, the dual-track process to gaining full citizenship renders impossible (Jitpiromsri et al., 2020; McCargo, 2011; McCargo and Thalang, 2023).

In addition to the identity and historical sentiment, structural inequalities and economic marginalization also contribute to the insurgency. According to the reports from various Thai NGOs, poverty and unemployment remain the biggest challenge. In 2023, three Deep South province GDP per capita remains the lowest among the southern provinces (Office of the National Economic and Social Development Council, 2024). Narathiwat is the lowest (64,005 baht), followed by Pattani (83,369 baht), and Yala (108,108 baht). The highest one is Phuket (314,921 baht). The unemployment rate in the southern border is the highest (2.4%), when compared with all Thai regions (Department of Employment, 2025). Especially among Malay youths, unemployment rate remains high, due to their education quality which relatively lower than the Thai Buddhist (Center for Conflict Studies and Cultural Diversity [CSCD] Men and Youth in Thailand’s Conflict-Affected Deep South, 2014). The government program, such as 4,500-baht employment project and the Graduate Volunteer programs, provide little impact on economic redistribution in the Deep South (deepsouthwatch, 2014). Therefore, the socioeconomic situation of Thailand’s Deep South revealed its structural inequalities, economic marginalization, which inflicted both conflict dynamics and peacebuilding efforts.

Research on the Malay Muslim identity in the Deep South began emerging in the late 1970s and since then has focused mainly on reciprocal action between the Thai state and insurgents (e.g., Suhrke, 1977; Scupin, 1980; Pitsuwan, 1987; Askew, 2008; McCargo, 2011). Such research has helped in providing the background of the Malay Muslim identity in Deep South. However, these studies have rarely focused on socio-cultural aspects of the Malay Muslim population and how the aspect links to the longstanding rebellion. Examples of the few such studies to do so are Suhrke (1977), Amir (2013), and Yamirudeng (2016). However, these studies scarcely touch upon aspects of Malay Muslim cultural resistance and the ethnic boundaries in Deep South. For example, available research on the Malay Muslim identity and insurgency has yet to provide adequate answers to question of from the perspective of ethnic boundaries, how significant the Malay cultural resistance to impede the Thai assimilation policies, which is critical for understanding the contestation of power and influence between Thai nationalism and Malay Muslim identities. In seeking to fill such gaps in the research, this study attempts to explore the Malay Muslim cultural resistance and their significance to the peace process in the Deep South.

This study employs Frederik Barth (1969) conception of ethnic boundaries. In his book Ethnic Groups and Boundaries, Barth argued that “ethnic boundaries” characterization is not limited to the traditionalist approach, such as the cultural content. Barth further elucidates his concept in two important ways. First, Barth argues the making of ethnic boundaries involves a process of social interaction, which is “social processes of exclusion and incorporation”. This social interaction makes ethnic boundaries can still be delineated, despite the dynamics within the ethnic group. To illustrate this further, the domestic migration of the Malay Muslim from Thailand’s Deep South to other provinces in Thailand could not diminish the Malay identity that person has. Secondly, Barth emphasize on “dichotomized ethnic statuses” for the maintenance of the ethnic boundaries. According to Barth (1969: 10), “cultural differences can persist despite inter-ethnic contact and interdependence”. Barth see the social process as a factor to define ethnic boundaries. The communication between groups could not threaten the existence of an ethnic boundaries. Yet, it will strengthen the sense of an ethnic community (Barth, 1969; Jakoubek, 2019, 2022; Jakoubek and Budilová, 2019; Siebers, 2017).

Barth concept is applicable to Malay Muslim population, as a group of people able to withstand and resist the influence of Thai nationalism. The concentration of Malay Muslims in the Deep South and their cultural resistance to partially reflects Barth argument. Barth’s assertions provide us with an understanding of why Thai nationalism do not manage to instill the Thai culture in all of Thailand territory. In Deep South case, a region dominated by Malay Muslim population with a cultural identity that differs significantly from that of the national majority. This study proposes that Barth’s conceptualization of ethnic boundaries helps explain why the Malay Muslim population of the Deep South has been the community most resistant to Thai nationalism when compared with other ethnic groups in Thailand.

This study, apart from the introduction and conclusion, consists of four sections. In the first section, we review existing research on the Malay Muslim identity in Deep South and discuss important gaps in the research regarding the role of Malay Muslim population’s cultural resistance. In the second section, we discuss the Malay Muslim cultural resistance as the part to the conceptual framework incorporating the work of Barth. In the third section, we discuss one case regarding Malay Muslim cultural resistance, namely the Melayu Raya Day, and we analyze how the Melayu Raya Day can be a starting point to better understanding the Malay Muslim ethnic boundaries, which will serve as a potentially useful contribution to the ongoing peace process.

## 2 Literature review

Research on Malay Muslim identity in the Deep South first began emerging in the late 1970s. Since then, there have been two main themes to this literature. The first theme concerns the Malay Muslim identity and the insurgency in Deep South (e.g., Srisompob and Panyasak, 2006; Feigenblatt, 2010). Srisompob and Panyasak (2006) research finds that ideological beliefs and social grievances are primary contributing factors to the insurgency. They also find that poverty is a precondition, while the ideology of local Malay Muslims comprises the impetus for the insurgency. Feigenblatt (2010) use the “small people” concept from Abulof (2009), to explore the” existential uncertainty” that the Malay Muslims face. The memory of the Patani kingdom has been gradually faded by the structural approach from the Thai government, such as omitting the Patani kingdom history in the Deep South. The Malay Muslim, who was previously independent, facing the Thai assimilationist policies, experience the gradual losing of their identity, which leads to the uncertain future of the Patani kingdom memories to its population.

The second main theme of research on Thailand’s southern insurgency concerns historical and cultural perspectives on Malay identity within the Thai nation (e.g., Scupin, 1998; Horstmann, 2007; Keyes, 2010; McCargo, 2009; Albritton, 2010). Most of the literature on this theme has been conducted by anthropologists, religionists, or culturalists. Scupin (1998) explores the reformation of Islam in various Thailand region including southern part. In the Deep South, the reformation contributes to the purification of Islam and the re-awakening of the Malay identity to against the assimilation from the Thai government, such as the transformation of Islamic traditional school, pondok, to the modern Islamic school. Horstmann (2007) argues that the field of anthropology is well-equipped to provide a clearer picture of the social issues faced by Malay Muslims amidst the conflict. Horstmann states that anthropology is a useful tool with which to create “local sovereignty” considering the historical and cultural values held by the Malays (Horstmann, 2007). Keyes’s (2010) research argues that non-Malay Muslims perceive Malay Muslims negatively due to the continuing instability of the situation in the Deep South, as well as largely negative mainstream Thai written and oral discourse (Keyes, 2010). Attacks by insurgents reinforce this discourse, which causes Malay Muslims to be profoundly alienated from larger Thai society. Duncan McCargo (2009) finds that politics of identity have caused a deterioration of religious tolerance in the Deep South (McCargo, 2009). McCargo finds that Thai Buddhists living in these provinces generally feel threatened as they are outnumbered by Malay in the area. Robert Albritton’s (2010) study argues that the political division in the Deep South is ethnocultural, “represented by orientations to Malay or Thai culture and identity, as indicated by the language spoken in the home” (Albritton, 2010).

Because the existing research on the Malay Muslim in Thailand’s Deep South has primarily been conducted according to these two general themes, the literature has focused on various actors and their roles, including the Thai government and military, insurgents, and the Malay Muslim population. The extant research, however, has mainly focused on the reciprocity between Thai state actors and the Malay Muslim population, leading to most academic discussion being entrenched around the narratives of identity and influence between the Thai state and various Malay Muslim groups. Therefore, the existing research on the Malay Muslim population in Deep South provides us with only a limited understanding of the ethnic boundaries of the Malay Muslim and how the ethnic boundaries could be an useful approach in addressing the Deep South peace process. With this current study, I have attempted to help fill the gaps in our knowledge about these issues.

The analytical framework for this research employs Barth (1969) conception of ethnic boundaries. Barth (1969) argues ethnic boundaries does not rely solely on cultural content such as language, ritual, or any other distinctive feature. Barth further argues a group’s ethnic boundaries is distinctive because it relies on social process, which is exclusion and incorporation. The exclusion and incorporation process contributes to a strong communal sense of cultural identity, which generates the capacity to resist cultural assimilation. To illustrate this further, The Malay Muslim population continues to practice and enact their cultural identity even after the Thai nationalism and modernization. The Malay Muslim remains unassimilated, since they incorporate the Patani kingdom memories into their culture. Moreover, the suppressing assimilationist policies from the Thai government, create the strong contestation between Thai nationalism and Malay Muslim culture. In the long term, this contestation is influential in creating the social exclusion, separating the ‘Thai’ identity from their ‘Malay’ identity.

The second way in which Barth has deepened our understanding of cultural resistance as the part of ethnic boundaries has been to provide new perspectives with which to examine the civilian Malay Muslim movement in the Deep South. The movement, in principle, aims to exercise the Malay Muslim culture and identity. However, this movement is also characterized by Barth second concept of ethnic boundaries, which is dichotomisation of culture. In this movement, the clear ethnic boundaries is drawn between Malay and Thai. The movement also will eventually become an important point when designing the sustainable peaceful conditions in the Deep South as the part of the peace process. Nonetheless, the existence of the movement is vulnerable to the Thai security agencies as they consider the movement as a part of the insurgency.

Barth’s exploration of ethnic boundaries supports this article argument: by a series of cultural resistance against the influence of Thai assimilation policy, the Malay Muslim community has managed to rigorously retain its ethnic boundaries vis-à-vis Thai national identity. First, Barth demonstrates that ethnic groups use their social process of exclusion and incorporation to affirm their ethnic boundaries, thus limiting the assimilation process. Second, Barth argued that there is no rigid ‘cultural border’. The dynamics of social process and communication, does not necessarily assimilate an ethnic group with other culture. For instance, a Malay Muslim remains as a Malay Muslim, even he or she speak Thai or any other languages. This is where Barth argument provides an important assertion: by the social process of exclusion and incorporation, cultural identity is not conceived as a rigid, unmovable nor changeable. In the context of the present study, I will examine the question of how cultural resistance as a part of ethnic boundaries making can be considered as a significant factor of Malay Muslim identity in the Deep South.

## 3 Research method

This article employs qualitative research, with two primary data collection methods; a short term observation and interviews (Creswell and Creswell, 2011). First, a short-term observation was conducted toward the Malay Muslim primarily on Pattani Province, from April 2022–May 2022. Additionally, field observation was conducted in the province of Yala and Narathiwat to diversify the observation and to compare the situation. In three provinces, the observation was conducted on urban and rural location. Hese observations is helpful to write the ethnography, by providing actual condition of the local people’s daily lives. Furthermore, the observation shows the knowledge, attitude, and practice of Patani Malay identity by the local population.

The second stage was by in-depth interview with a total of 14 key informants. Of these 14 key informants who agreed to participate in this study, 2 were university lecturers, 1 was an NGO worker, 1 was a Malay student organization member, 4 were Islamic teacher, 1 was an history teacher, and 4 were local Malays. All informants were selected based on the following criteria: broad knowledge and familiarity with issues relevant to this research (Thai assimilation policies and Malay cultural resistance), representation of diverse communities, and residents of the research site who have lived experience with the insurgency. The interview employs a semi-structured, casual, conversation-like style to allow the key informants to share information as they felt comfortable. A focus group discussion (FGD) was conducted with three school teachers in Yala Province. The primary language used during interviews was Patani Malay interspersed with certain words from central Thai. However, in cases when informants were fluent and comfortable using English, interviews were conducted in English, particularly with non-Malay Muslim interviewees.

Verbal informed consent was obtained from all participants during data collection, and issues of safety and confidentiality were prioritized throughout the research process. Given that some fieldwork was conducted in areas under martial law, particular attention was paid to risk mitigation, including careful selection of interview locations and procedures. All participants were fully informed about the aims of the study, and interview questions were tailored to each participant group to ensure appropriateness, security, and privacy. Contact with all informants was maintained following the interviews in case additional data was required. In order to maintain participants’ privacy, interviews were voice-recorded only after receiving consent from each informant, after which each interview was transcribed with all names and sensitive, identifying information redacted. Pseudonyms are used for all informants in this research in order to protect their privacy and security.

Furthermore, the data analysis technique mainly relies on the discourse analysis from the interview and the ethnography. To triangulate the primary data, the secondary data (journal, books, news) is used as well. The reason to use the secondary data is to reduce the possibility of primary data misinterpretation (Creswell and Creswell, 2011).

## 4 Finding and discussion

### 4.1 History of the assimilation of Malay Muslim

After Patani became embroiled in a series of wars in the southern part of Thailand between 1767 and 1786, Siam finally succeeded in conquering the Patani Kingdom in 1786. Soon after, the Siamese government began implementing assimilation policies for the region’s Malay Muslim population. The aim of assimilation was to deepen the sense of “Thainess”—a term coined by Thongchai (1994)—in the region in order to reduce the potential for future dissent. The local Malay population regarded these policies as attempts toward “Siamization” and vigorously resisted these (Ai-Sen, 2016; Arphattananon, 2018; Ayuttacorn et al., 2023; Praphan, 2026; Thongchai, 1994; Wekke et al., 2019; Yong, 2023a). Another reason for the discontent of the Malay Muslims with Thai assimilation policies is due to certain of their elements. For example, Buddhism is strongly embedded in the meaning of “Thainess,” leading to the conception that ‘being Thai means being (Albritton, 2010; Carter, 2020; Praphan, 2026; Thongchai, 1994; Yusuf and Thongin, 2025).

During the administrations of PM Phibun Songkhram (1938–1944, 1948–1957), the Thai government issued 20 cultural mandates collectively known as Ratthaniyom. The Ratthaniyom dictates cultural expressions and norms that are considered suitable and appropriate for Thailand’s national identity. For example, Thai national dress was dictated to follow European clothing styles and standards, while forms of Thai traditional dress and Malay clothing were banned and considered incompatible with the national identity (Ai-Sen, 2016; Amir, 2013; Charoenvattananukul, 2022; Forbes, 1982; Jory, 2007; Yoshikawa, 2011) Malay Muslims perceived the Ratthaniyom as an additional oppressive measure of the Thai state’s assimilation policies. The Malays themselves reject these mandates because they consider ‘Thai’ to be strongly associated with Buddhism, while the term ‘Malay’ shares connotations with Islam (Munirah, 2018). The label ‘Thai Muslim’ that is imposed by the Thai government to them is interpreted by the Malay Muslim community as equivalent to being referred to as ‘Buddhist Muslims’, which is unacceptable to them. The imposition of the label ‘Thai Muslim’ (or ‘Thai Islam’) is only one aspect of Thai nationalism to conflict with the local Malay Muslims’ identity, contributing to the Malay Muslim population’s cultural resistance to nationalist Thai values (Arphattananon, 2018; Chalermsripinyorat, 2020; Jory, 2007; Lamey, 2013; Yong, 2023b).

The struggle to defend Malay identity from threats of Thai assimilation began in earnest in the 1940s. The first such movement was initiated in 1948 when Haji Sulong, an Islamic scholar widely respected among the Malay community, advocated for Malay culture and Islamic law privilege in the three southernmost provinces of Thailand through a seven-point decree. However, the Thai government gave a mixed response to his demands, accepting some while rejecting others (Ockey, 2011; Yong, 2014, 2023b) Similar demands were more recently expressed by BRN. The BRN demanded sovereign rights (hak pertuanan) in a video posted to YouTube on April 26, 2013 (Muhammad, 2013).

Since the insurgency begin, neighboring countries gave a mixed response to the insurgency in Deep South. Malaysia as a country which both geographically and historically close to the Deep South has a particular role. The Malay Muslim community usually has relatives living in northern Malaysia (Funston, 2010, p. 236). In this sense, Malay transnationalism can be seen, which contribute to resilience. To begin with, the first negotiation between Thai government and the insurgents took place on Langkawi Island in Malaysia in 2006, with Malaysia as the mediator. BRN as insurgent groups also emphasize Malaysia as the mediator, proven by the YouTube video released by BRN on 26 April 2013 (Muhammad, 2013). Following the negotiation in 2006, there were some initiatives arranged by the third party, such as the Bogor talks in Indonesia in 2008, the Organization of Islamic Conference in 2010, and the Center of Humanitarian Dialog (HD Center) in Bahrain in 2007 (Chalermsripinyorat, 2020, pp. 143–147). Indonesia in this case also express its concern to the situation in Deep South, yet adhere to the ASEAN non-intervention principle (Yusuf, 2007). The Organization of Islamic Conference (OIC) also see the possibility of solving the problems from both Thai and Malay perspective (Yusuf, 2007).

### 4.2 Ethnic boundaries

As noted above previously, Barth (1969) conceives of ethnic boundaries are not merely made from the distinctive cultural identity, but also involves a social process. We suggest that, in the case of Malay Muslim cultural resistance in Thailand’s Deep South, the Malay Muslim community has extensively exercised the character ethnic boundaries, and the following two case support our argument.

The first case concerns the political movement. One prominent manifestation of the political movement has been the creation of the Wadah, with the objective of integrating Malay political elites into the national Thai government. The Wadah is a progressive platform established in 1988 by Malay Muslims and was initially led by Den Tohmeena, son of the highly respected local Muslim leader Haji Sulong. The Wadah aims to elevate issues and concerns of Malay Muslims, such as the security situation in the Deep South, to the national level of Thai politics (Connors, 2006; Utarasint, 2019).

Presently, key members of the Wadah are represented in the Thai political party known as the Prachachart Party. These days, Prachachart has become well-known as the pan-Muslim party in Southern Thailand (Chachavalpongpun, 2023; Chambers and Supajakwattana, 2025; McCargo and Thalang, 2023; Utarasint, 2019). The leader of the party, Wan Muhammad Noor Matha, who also serves as the speaker of the government house, claims his party is an alternative for voters across the country. In the 2023 national general election, all the seats won by the Prachachart Party were in the Deep South (three in Pattani, three in Yala, and one in Narathiwat) (Thai PBS, n.d.). The election results reveal the trust from the Malay Muslim to the political movement made by the party. However, the party is only popular at the three provinces. Silk (an NGO member) said, “So, they won only in this region. So, it might be called local parties for Malay Muslim, but because of the requirements of parties, they have to send the candidate to other regions as well. This is difficult.” (Personal Interview, 2022). In addition, the emergence of a new popular party among youth, Move Forward Party (MVP), create a divergence of opinon. For the youth, MVP strong opposition on military control and the chance of reforming the monarchy control is attractive. Furthermore, McCargo and Thalang (2023) wrote, “They were mistrustful of rhetorical appeals to identity issues, especially comingfrom Prachachart politicians with a long track record of discreditable collaboration withthe Thai state” (McCargo and Thalang, 2023, p. 643).

The establishment of the Malay Muslim political party was characterized by the social process of exclusion and incoporation. The party major victory in the 2023 election displays the Malay Muslim acceptance to the party in Deep South. The agenda of the party also incorporates the Malay Muslim demand, such as the expansion for Islamic and other religious education (Larsson, 2023). This agenda provides two direction. First, it reveals the party intention to defend the Malay Muslim ethnic boundaries. Second, the party has also shown its dedication to create a path toward the multicultural society. Eventually, these two directions provide a win-win solution for both Thai government and ethnic minorities in Thailand, including the Malay Muslim. Nonetheless, the idea of multicultural society is not something new. A plan from an official in charge in the Ministry of culture in 2003 endorsed the multiculturalism. In 2004, this plan emphasized on culture as ‘capital’. This plan indicates the Thai government gradual change of vision, from upholding “Thainess” to be a country which respect the existence of other ethnics (Hayami, 2006). A secretary-general from Southern Border Provinces Administrative Center, Tawee Sodsong, also bring the multiculturalism approach when working together with Prachachart Party (McCargo and Thalang, 2023). Therefore, creating a chance toward the better freedom of expression of Malay Muslim.

The second case concerns the Malay Muslim civilian movement. The cultural resistance by the Malay Muslim display the characteristic of ethnic boundaries, which is to socially exclude themselves apart from the Thai national identity. In doing so, they established multiple civilian movement. Historically, the civilian movement such as Gabungan Melayu Patani Raya, or the Greater Patani Malay Association (GEMPAR), Civil Society Assembly for Peace (CAP), and the Federation of Patani Youth and Students (PerMAS) actively advocate for the peace process and promote Malay culture and identity. Since 1940’s until today, they have demonstrated their commitment to create and maintain their boundaries from being assimilated. These civilian movements in its principle aims to create the Malay identity exist without being compromised by the Thai assimilation policies (Panjor, 2018; Suciyani and Mamaeng, 2024).

Malay Muslim cultural resistance also display another characterisitic of ethnic boundaries, which is “dichotomized ethnic status”. There are two ways. First, the resistance asserts the self-ascription of being a ‘Malay’ in the Deep South. As a Malay, the resistance make a clear dichotomization between the Thai and Malay identity. Furthermore, ‘Malay’ and ‘Muslim’ for those Malay Muslim in Deep South are inseparable and contributing to ethnic boundaries. The resistance affirm these two elements are established, which to maintain a clear definition of ‘Malay Muslim’, which also reveals the separating line between Thai Muslim and Malay Muslim. Second, the process of dichotomisation is apparent on Malay Muslim historical perspective of Thailand or Siam (Jory, 2007).

A deep explanation was provided by Flight (an university lecturer), “They never admit themselves to be proud as Siamese. Siamese has their king, but they (Malay) are not proud of it. They have no pride in it (the king). I could say their Malay identity is getting stronger. Probably, on the lower (social) layers, they still have the intention to defend their identity” (Personal interview, 2022). Maroon (an university lecturer) further explain why the Malay and Muslim are inseparable, “For the Malay people here, they don’t like this word, Siae (Siam) Malay. Because the two words are contradicted. How they become a Malay, and at the same time become Siae (Siam)? Because for them the word Siae means Buddhist. So, it cannot go together” (Personal interview, 2022). The statement from Flight and Maroon also exemplify the Malay Muslim reluctance to genuinely adopt the Thai national identity—let alone their submission to the King. Another interpretation from these two key informants is the Malay Muslim are reluctant to abandon their Patani kingdom memory from their mind. For them, the kingdom may fall, but the identity shall never be erased.

Notwithstanding the insurgent’s perspective that the Thai culture is a threat to the Malay Muslim identity, the Malay Muslim does not perceive the Thai culture per se as a threat to the Malay Muslim identity. Based on the fieldwork interview, the characteristic of ethnic boundaries such social process of exclusion and incorporation is applicable for the Malay Muslim. For instance, the harmonious life between three ethnics (Chinese, Thai, and Malays) in Pattani province prior to the escalation of the insurgency in 2004 give an example of how the three ethnics could co-exist without compromising each other’s culture. Nathan (a Permas member), said, “In the past, before the year ‘47 (2004), Chinese, Buddhist, and Malay here can make friend. They can eat together. But after ‘47 (2004), not anymore” (Personal interview, 2022). Susie (university worker), said, “The Buddhist and Muslims, do not have much conflict, they don’t have any problem with each other” (Personal interview, 2022). Mick, a history teacher, expressed the same, “In fact, the history between Siam and Pattani is peaceful, very peaceful. The (Pattani) country that has a strong economic hub (in the past), is Siam”(Personal interview, 2022).

The local Malay perspective reflects the practice of Barth’s ethnic boundaries: to create each other culture space, living harmonious and without any threat to assimilating each other. On a textual basis, the phrase like ‘they can eat together’ (Nathan), ‘do not have much conflict’ (Susie) indicate the practice of ethnic boundaries through social process have been done long before the insurgency in 2004. For the Malays, the problem only exists when the state enforces or directly encourage to use Thai identity for them. Flight (university lecturer) said,” The people in Patani have a “hard culture,” in which to adjust themselves to larger societies such as the Thai majority, they could not enter (the society), and they have difficulty assimilating” (Personal interview, 2022). When the assimilation policies in the past affect the Malay Muslim, the trace is still visible today.

### 4.3 The Melayu Raya day case

**Figure 1:**
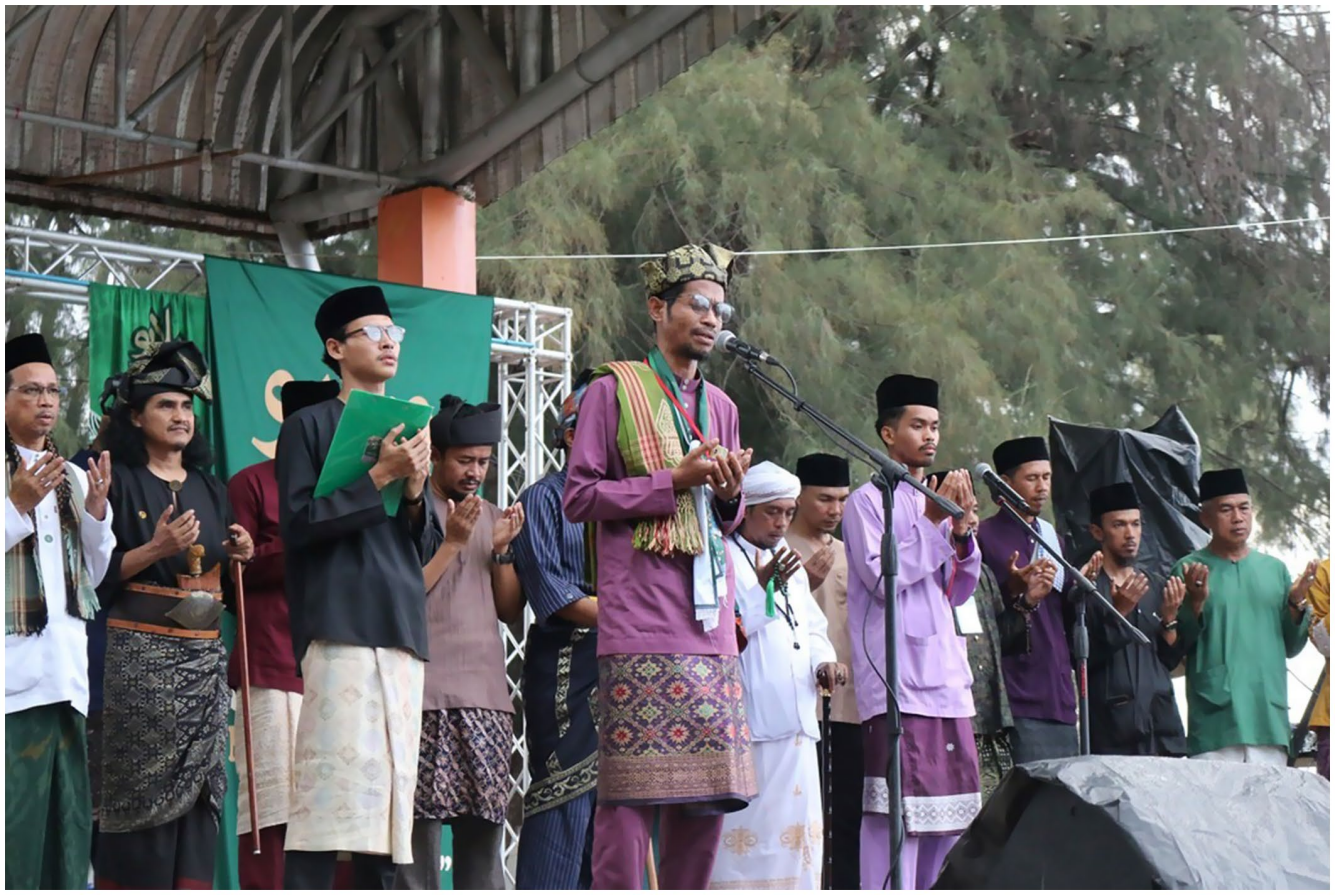
Melayu Raya event. Source: Prachathai.com (Published 3 April 2025). Licensed under CC BY-NC.

One of the well-known Malay cultural resistance events is Melayu Raya day, which is also known as The Great Malay Assembly (Perhimpunan Melayu Raya). The Melayu Raya day is part of a campaign begun in 2014 and led by the Civil Society Assembly for Peace (CAP). CAP aims to reintroduce traditional Malay clothing following the previous ban on such clothing by the central Thai government. According to Anattata Naser, founder of The Great Malay Assembly, the event is intended to reignite local Malay cultural spirit amidst the Thai government’s efforts to homogenize all Muslims under the diminished identity ‘Thai Muslim’ (Iziddin, 2023; Figure 1).

The first Melayu Raya day was held in 2019 at the historical Krue Se Mosque in Pattani Province. However, the COVID-19 pandemic caused the event to be canceled in 2020 and 2021. In 2022, the assembly resumed and took place on a beach in Saiburi (which local Malays refer to as Teluban), Pattani Province. One possible reason to do the 2nd event at the Teluban beach is due to the size of the participants. For example, in both 2022 and 2023, more than 30,000 Malay Muslims—mostly youth—attended the event. In 2024 and 2025, the number increased to be almost threefold where a Malay Muslim-based social media, Wartani, reported 80,000 Malay Muslims attended the event (Iziddin, 2023).

The Melayu Raya day caries a different theme annually. The theme ranges from Malay culture, solidarity to Palestine (Deurahem, 2024), and the latest one in 2025 carries the theme *Green Melayu, Suci Patani* (Green Malay, Patani Purified). The assembly is typically held on the third day of the Eid Fitr celebration. Due to Islamic tradition, which discourages mixed-gender gatherings, event activities are organized separately for males and females. The event for females is made few days after the day for male.

The Melayu Raya day is perceived as a suspicious activity from the Thai government. It is not surprising the Thai government view Melayu Raya day as a suspicious activity. Because they see the participant, rightly or wrongly, as associated with groups that are sympathetic to the insurgency. The event, moreover, implicitly bring a certain degree of ‘Malay ethnonationalism’, in a Thailand-controlled territory. For instance, on the day, the Malay youth brought the flag of their village, some Malay-speaking countries flag like Indonesia, Malaysia, or Brunei. To some extreme case, some even brought the BRN flag, which is also the basis of the Thai government suspicion (Sattaburuth, 2024; Deurahem, 2025).

On 14 December 2023, the Thai government issued Strategic Lawsuits Against Public Participation (SLAPP) against 9 Malay cultural activists and organizers of the event (Bangkok Post, 2024). It is commonly known that SLAPP-related lawsuits have sparked controversy among the general Thai public as they have also been used to disrupt the work of civilian activists in other areas of the country (Sattaburuth, 2024). The allegation was made by the police that the activists have created a “public disturbance to the national stability” on the Melayu Raya day in 2022. The police called them to go to Pattani Provincial Police on 9 January 2024. On 3 October 2024, the 9 activists submitted a request for justice to the Office of the Attorney General, to review their case. The office responded on 7 January 2025, by an order to terminate the justice request (Nanuam, 2024).

As a parallel approach for the justice to the 9 activists, the Malay Muslim population began a crowdfunding with the name *Tabung Keadilan Patani* (Patani Justice Fund). This fund is given for those who are prosecuted exercising the right of freedom of expression in the Deep South. Finally, on 23 January 2025, the 9 activists were bailed out with the cash bail of 70,000 Thai Baht per person, or 630,000 Thai Baht in total. 350,000 Thai Baht from the Patani Justice Fund and 280,000 Thai Baht from the People’s Fund (tlhr2014.com). On 27 February 2025, United Nations Special Rapporteur also issue an official letters and statements concerning the Thai government criminal charges leveled against the 9 activists (srdefenders.org). On 29 July 2025, the case of Melayu Raya day is still at the court where the 9 Malay Muslim activist needs to be investigated regarding the activities done on the Melayu Raya day, and the clarification regarding it. The investigation process is scheduled until August 2025.

Consequently, the following Melayu Raya days from 2023 onward were organized under the permission of local Thai security forces, including the local administration office (Iziddin, 2023). Since 2024, a Thai security agency which commonly known as Internal Security Operations Command Region 4 or ISOC 4, co-hosted the event, which to ensure similar incident like in 2022 will not happen again. For instance, “no flags that may pose security risks are allowed at the event, except for village or club/organization flag” (issoc4news.blogspot.com, 2025) (ISOC, 2025; ISOC 4).

**Figure 2:**
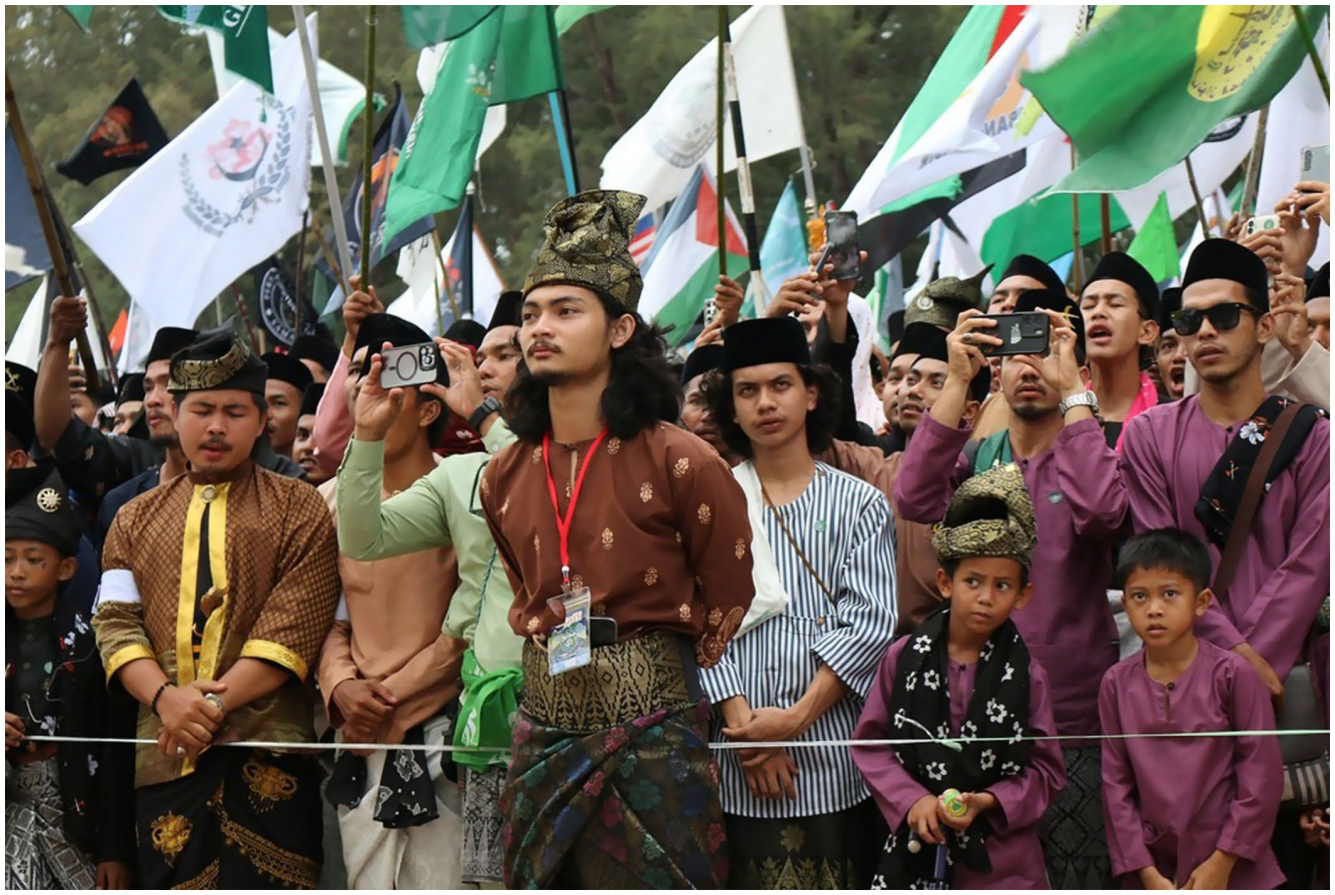
Melayu Raya participants fully dressed in malay clothing. Source: Prachathai.com (Published 3 April 2025). Licensed under CC BY-NC.

The Melayu Raya case was characterized by the ethnic boundaries in two ways. First, the case displays the process of social exclusion and incorporation. The Melayu Raya day incorporate all Malay—mostly youth—to exhibit their Patani Malay heritage. The ‘Patani’, which historically belong to the Patani Kingdom, covers 3 provinces (Pattani, Yala, Narathiwat) and 4 districts in Songkhla province (Thepha, Chana, Saba Yoi, Na Thawi and Sadao) (Lamey, 2013). Regardless of their home province, they come to unite as one at the Teluban beach. The incorporation of Malay identity is apparent here, as instanced on Figure 2.

**Figure 3:**
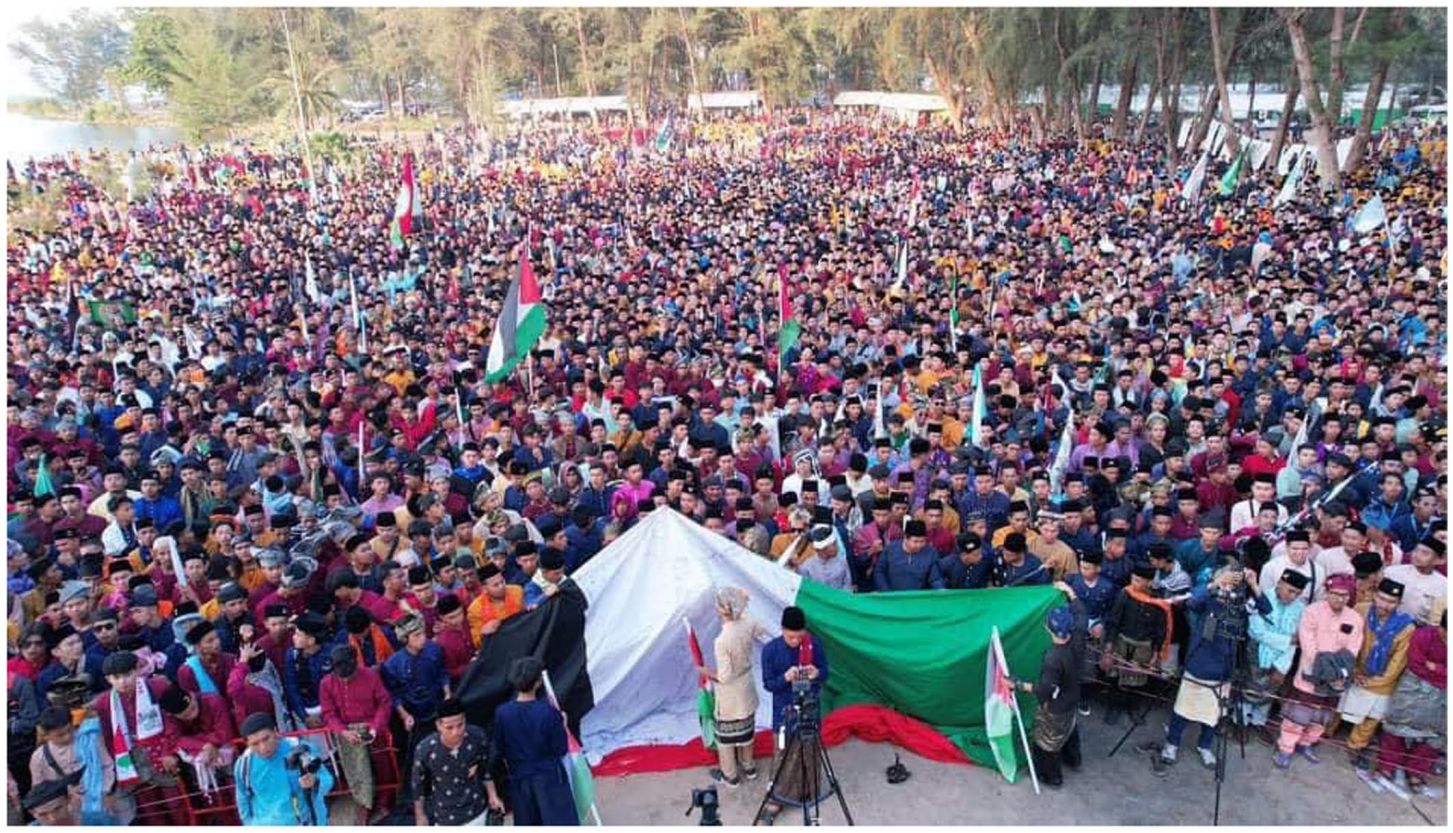
A crowd of Melayu Raya participants fully dressed in traditional Malay clothing. Source: Prachathai.com (Published 14 April 2024).

**Figure 4:**
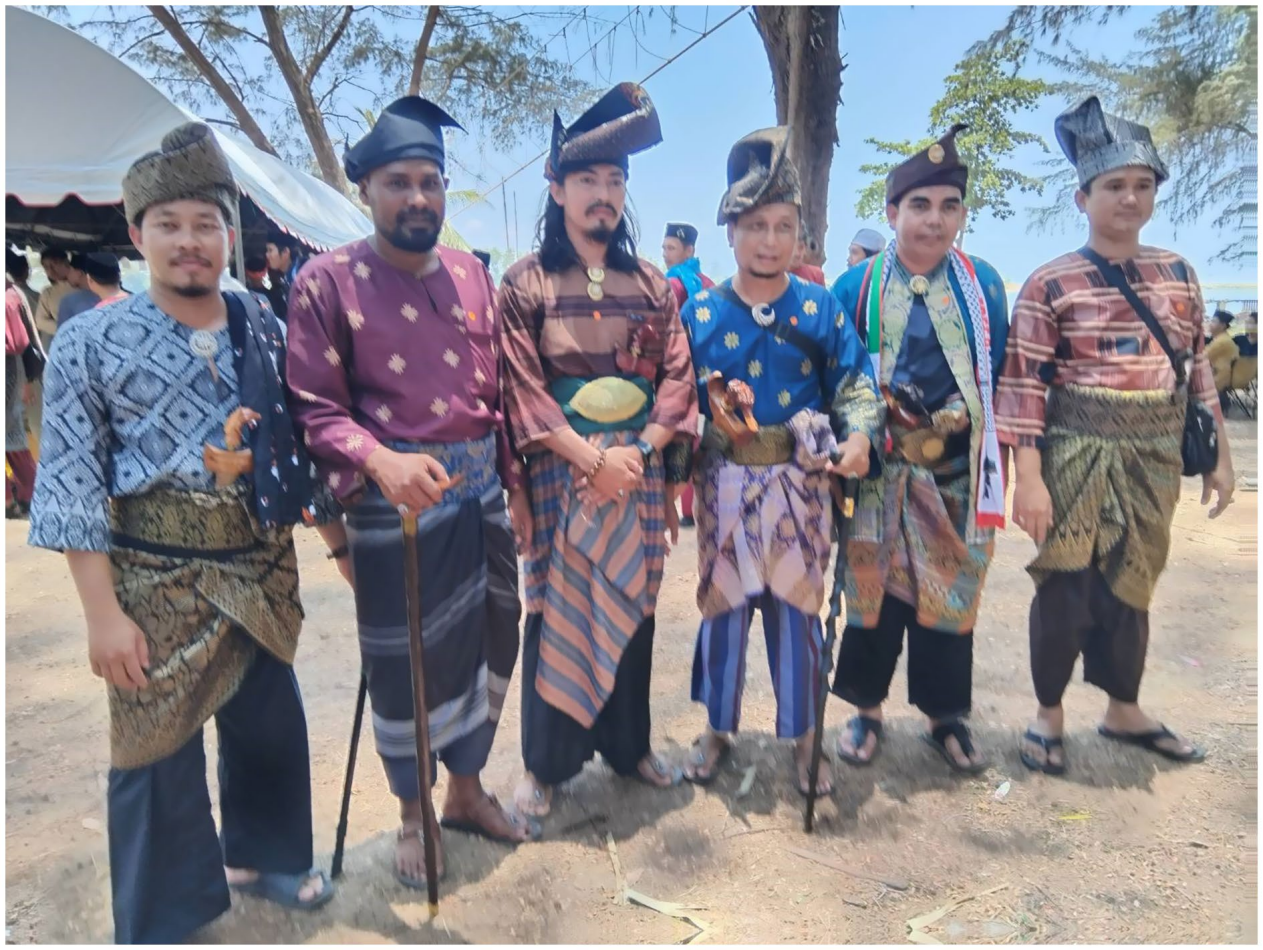
A demonstration of Malay traditional clothing, including keris on the waist. Source: Prachathai.com (Published 14 April 2024). Licensed under CC BY-NC.

**Figure 5:**
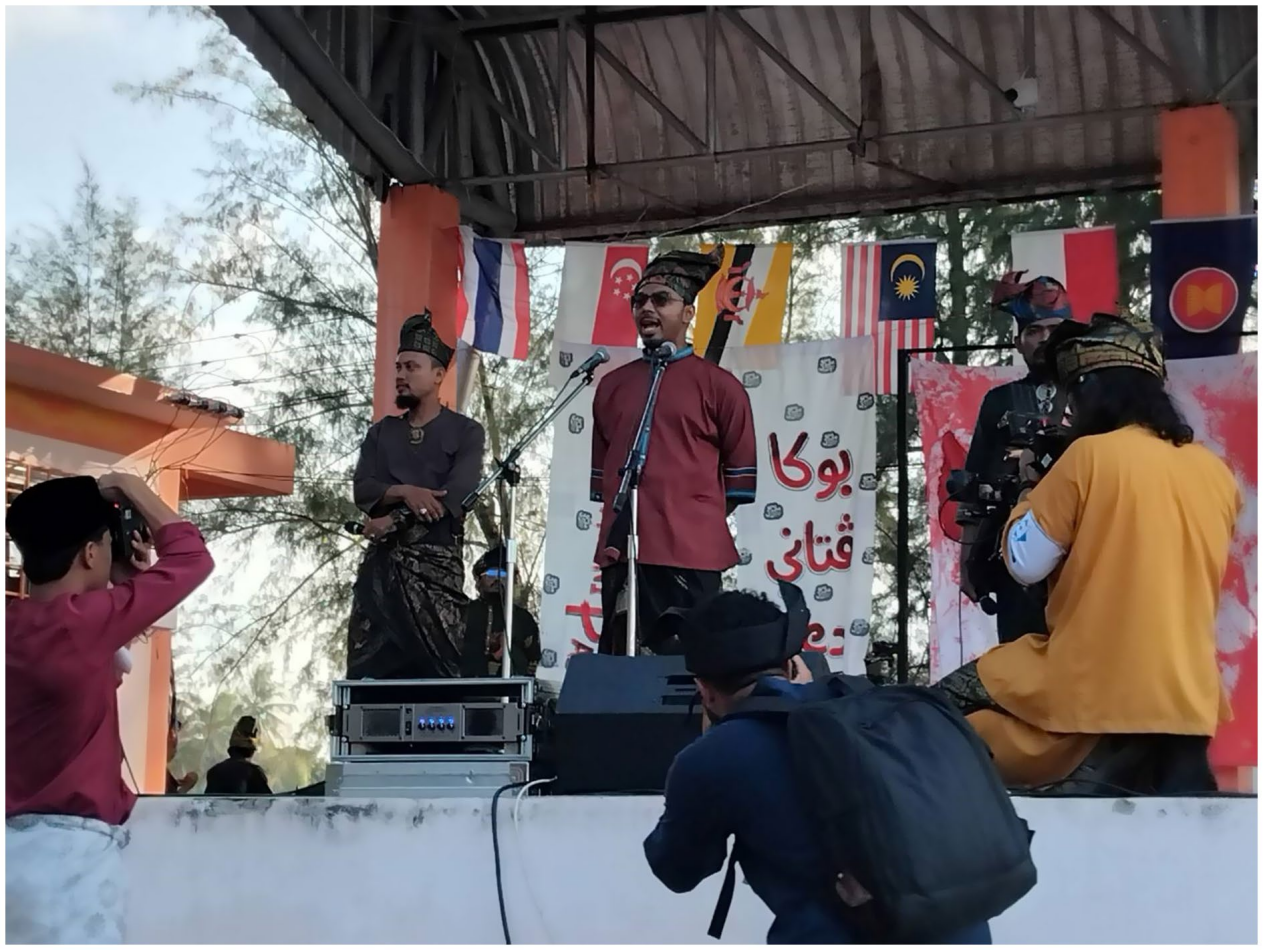
Demonstration of Malay language written in Jawi script, behind the speakers. Source: Prachathai.com (Published 14 April 2024). Licensed under CC BY-NC.

Second, the Melayu Raya day has demonstrated the characteristic of ‘ethnic boundaries’, in the way that the Malay youths are publicly dichotomise their Malay Muslim identity from other culture, including Thai Muslim and Buddhist. In relation to this ethnic dichotomisation, Sally, a female islamic teacher said, “Unlike in the past, promoting Malay writing was difficult, people were afraid, either on Facebook, or Instagram, they were afraid. However, now, praise god, because many people know their identity, that we are Muslim with Malay culture. Don’t be silent, because if we keep silent, our language or our culture and customs will disappear” (Personal interview, 2022). From the socio-cultural perspective, Maroon (an university lecturer) emphasized more on the interlinkage of Malay and Islam, “But here, in southern Thailand, to be Malay is to be Muslim, to be Muslim is to be Malay. As an example, if someone convert to Islam, they will not say to him or her, convert to Islam. They will say “masuk Melayu*” (entering the Malay). Enter to the Malayness*” (Personal interview, 2022; Figures 3–5).

Barth’s conception of ethnic boundaries can provide a guide in search of factors that explain Malay Muslim cultural resistance in Deep South. Two factors look promising in this regard: the Malay Muslim cultural resistance use ethnic boundaries characteristics to (1) to against in Thai assimilation policies, and (2) to define the clear history and boundary between the Malay Muslim culture and the Thai culture. Nonetheless, the dynamics of life of Malay Muslim is also influential in shaping the ethnic boundaries. Through these and the current cases, alternatives and solutions for the peace process can be provided.

## 5 Conclusion

The modern insurgency in the Deep South has further demonstrated a contestation over identity between the central Thai government and the Malay Muslim population. Historical incidents such as the subjugation of the Patani Kingdom and the government’s more recent assimilation policies have created deep divisions between Thai Buddhists and Malay Muslims.

This article assert that the Thai government’s assimilation policies have compelled Malay Muslim to define their ethnic boundaries by cultural resistance. There are two case that support this argument. The first case is the formation of political movement such as Wadah. The second case is civilian movement, such as Melayu Raya day. The day demonstrate the Malay Muslim commitment to retaining their Malay ethnic boundaries amidst the force of Thai assimilation policies. Despite these cultural preservation efforts, the actions of Thai security agencies, including SLAPP against the 9 cultural activists, have impeded the Malay Muslim struggle for broader recognition and acceptance of their identity.

This article is intended to contribute to the field of peace studies, particularly to our understanding of the peace process in the Deep South. This research also adopts a new approach by utilizing ethnic boundaries as an approach with which to better understand the contributing factors of an insurgency. The voices of key informants and focus group participants in this article amplify the perspective of grassroots, civilian members of society who reside within the conflict zone. This research also captures the essence of Malay Muslim cultural resistance and the lived experience of civilian activists going about their daily lives under martial law in Thailand’s three southernmost provinces.

This research is not without limitations. Our inability to travel within some areas in the three provinces due to security concerns hampered my ability to speak with a more diverse range of interviewees. As martial law was still in effect during the period of data collection, concerns for my personal safety as a foreigner in Thailand also imposed limitations. Challenges due to lack of time and potential lack of trust from the standpoint of interviewees, particularly government officials, created other limitations. For example, although the first author made attempts to contact them, he failed to secure an interview due to a lack of trust. However, utilizing a qualitative research approach with interviews from multiple actors has contributed much to the robustness of the findings and data diversifications, thus achieving the initial objective in undertaking this research.

Based on the findings, the authors would like to provide 3 actionable suggestions for both Thai government and Malay Muslims population. First, providing common ground for both sides to initiate a mutual understanding from both the Thai and Malay factions. Second, providing spaces for exercising the Malay identity, such as the creation of the Patani Malay language institution, eliminating the Thai security agencies pressure on freedom of expression by the Malay Muslim. Nonetheless, a fair and just treatment also need to be implied for other ethnicities to retain a fair, harmonious co-existence between Chinese, Malay Muslims and Thai Buddhists in southern Thailand. Third and last, stimulating an economic growth in Pattani, Yala, and Narathiwat. For instance, investment in several areas, targeting the rubber agriculture and fisheries can minimize the unrest among Malay Muslim population. Domestic economic incentives can also help to reduce the conflict since the Malay Muslim population can have upward social mobility without being afraid of losing their Malay identity.

As this study introduces a new approach in illuminating connections between insurgency, civilians, government institutions, and ethnic identity, further study is required to more deeply explore the social interaction between Thai Buddhists and Malay Muslims in the Deep South. Insight into the dynamics of interaction between these two groups could likely provide further understanding into multiculturalism and armed insurgency, as the casualties have come from both ethnic groups. Moreover, understanding the dynamics interactions would enable a proposal to the Thai government could take to address the insurgency in a more balanced manner in order to achieve a mutually beneficial resolution. An exploratory approach to the economic situation in the Deep South is also encouraged, as solving issues of poverty and unemployment in the region has the potential to encourage the engagement of insurgents in peace negotiations, leading to greater stability and co-prosperity in Southern Thailand.

## Data Availability

The raw data supporting the conclusions of this article will be made available by the authors, without undue reservation.
